# Long-Term Exposure to Inflammation Induces Differential Cytokine Patterns and Apoptosis in Dendritic Cells

**DOI:** 10.3389/fimmu.2019.02702

**Published:** 2019-11-21

**Authors:** Laura Stentoft Carstensen, Olivia Lie-Andersen, Andreas Obers, Michael Douglas Crowther, Inge Marie Svane, Morten Hansen

**Affiliations:** ^1^National Center for Cancer Immune Therapy (CCIT-DK), Department of Oncology, Copenhagen University Hospital Herlev, Herlev, Denmark; ^2^Department of Bioengineering, Technical University of Denmark, Lyngby, Denmark; ^3^Immunitrack ApS, Copenhagen, Denmark

**Keywords:** dendritic cells, inflammation, apoptosis, IL-12p70, IL-10

## Abstract

The activation of dendritic cells (DCs) has profound implications and governs the control of adaptive immunity. However, long-term activation might drive exhaustion of immune cells and negatively affect functionality. Here, long-term vs. short-term exposure to bacterial lipopolysaccharide and interferon (IFN)γ was evaluated on human monocyte-derived DCs. Long-term activated DC1s began to undergo apoptosis concomitant with a profound TAM-receptor and efferocytosis-dependent induction of interleukin (IL)-10. Whereas, levels of IL-12p70 and IL-10 were positively correlated upon short-term activation, an inverse association occured upon long-term activation and, while short-term activated CD1a^+^ DCs were main producers of IL-12p70, CD1a^−^ DCs were the main fraction that underwent apoptosis and released IL-10 upon long-term activation. Moreover, pre-apoptotic long-term activated DCs were no longer able to activate alloreactive IFNγ-responsive T cells present in peripheral blood mononuclear cells from healthy volunteers. The IFNγ response was mediated by IL-12p70, as a strong reduction in IFNγ was observed following blockade with an IL-12p70 neutralizing antibody. Finally, multiplex analysis of DC supernatants revealed a particular pattern of proteins associated with apoptosis, cancer and chronic inflammation partly overlapping with gold standard DCs well-known for their inability to secrete IL-12p70. In conclusion, long-term activated DC1s significantly changed their profile toward a non-functional, tumor-promoting and anti-inflammatory phenotype.

## Introduction

Dendritic cells (DCs) are antigen-presenting cells that primarily process and present antigens to T cells. As such, DCs bridge the innate and adaptive immune system and play a vital role in generation of both immunity and tolerance (1). Immature DCs are specialized in antigen uptake and processing. They are activated by pathogen-derived molecular patterns or danger-associated molecular patterns on dying, stressed, or injured host cells resulting in inflammation and perturbed tissue homeostasis (2). Lipopolysaccharide (LPS) constitutes a classical pathogen-derived molecular pattern activating DCs via Toll-Like receptor 4 (3). Strong synergy is provided by interferon (IFN)γ released from e.g., innate lymphocytes such as NK, NKT, or Gamma Delta (γδ) T Cells driving release of inflammatory cytokines including interleukin (IL)-12p70 (4), which control IFNγ-dependent adaptive immunity in conditions of viral infection and cancer (5). However, long-term exposure to mediators of acute inflammation might signify a transition into chronic inflammation and could be detrimental in the context of tumor-specific adaptive immunity. As such, strong evidence links inflammation to development of cancer, primarily involving the transcription factor nuclear factor-κB and signal transducers and activators of transcription 3 (2).

As an *in vitro* model, monocyte-derived DCs (MoDCs) are relevant counterparts of monocyte-derived inflammatory DCs found *in vivo* during disease-associated conditions of inflammation whereas current models are less optimal at studying homeostasis (6, 7). Upon activation, MoDC derived cytokines such as tumor necrosis factor (TNF)α, IL-6, IL-10 and IL-12p70 plateau from about 12–48 h post activation with LPS alone or LPS plus IFNγ (8). Looking at mRNA levels, TNFα and IL-6 peak around 3 h followed by IL-12p35 and p40 around 8 h and IL-10 around 18 h. Intracellular cytokine staining confirm that IL-12p70 is produced around 18 h as opposed to 42 h post activation (9) thus concluding that cytokine production halts approximately 24 h after initiated activation. To our knowledge no other studies compared the phenotype and functionality of long-term (>48 h) vs. short-term activated MoDCs. In contrast, several studies compared various types of activation cocktails comprising either inflammatory mediators alone, most prominently the gold standard DC cocktail (sDC; TNFα, IL-1β, IL-6, and PGE_2_) (10) or cocktails employing a mixture of Toll-Like receptor agonists combined with interferons (11–13). In addition, it is described how MoDCs constitute a heterogenous population of cells most profoundly as examplified by CD1a^+^ and CD1a^−^ subfractions (7, 14).

Long-term activation of MoDCs could be a relevant model to study the *in vivo* situation of chronic inflammation elicited by viral infection or cancer. To test this, MoDCs were activated with LPS and IFNγ (Type 1 DCs; DC1) for 1 (18 h), 2, 3, or 4 days, and compared with immature or 18 h activated gold standard (s)DCs. We performed extensive analysis on DC culture supernatants and tested the ability of long-term vs. short-term activated DCs to induce alloreactive cell activation. Long-term inflammatory activation caused a striking change from pro- to anti-inflammatory cytokines concomitant with induction of DC apoptosis and inability to activate alloreactive IFNγ-responsive T cells. Furthermore, a striking pattern of proteins associated with apoptosis and chronic inflammation partly overlapped with sDCs well-known for their inability to secrete IL-12p70.

## Materials and Methods

### Media and Reagents

All cell cultures were performed in complete medium consisting of RPMI 1640 medium supplemented with 10% fetal bovine serum and 1% penicillin/streptomycin (all from Gibco). Cell cultures were maintained at 37°C in a humidified atmosphere of 5% CO_2_. The following reagents were used for generation and activation of DCs: Recombinant human GM-CSF (PeproTech), IL-4 (PeproTech), IFNγ (PeproTech), TNFα (PeproTech), IL-1β (PeproTech), IL-6 (PeproTech), LPS from *Escherichia coli* serotype O55:B5 (Sigma-Aldrich) and PGE_2_ (Sigma-Aldrich).

### Generation and Activation of DCs

Monocyte-derived immature DCs were either produced in house from buffy coats or were optained as a kind gift from M.W. Pedersen and H.B. Grav, Symphogen A/S, Denmark. Immature DCs were generated from monocytes purified from PBMCs by positive selection employing CD14-reactive magnetic-activated cell sorting beads (Miltenyi Biotech). After 4–7 days in culture with 20 ng/mL GM-CSF and IL-4, iDCs were harvested by vigorous pipetting, washed and stored at −140°C. In select experiments, iDCs were stained for CD1a expression and sorted into CD1a^+^ and CD1a^−^ fractions on a MACSQuant Tyto Cell Sorter (curtesy of Olof Berggren, Miltenyi Biotec). Prior to activation, thawed iDCs were rested for 1–4 days in complete medium with 20 ng/mL GM-CSF and IL-4 in 24-well plates in 1 mL/well at a density of 0.5 × 10^6^ cells/mL. In selected experiments, iDCs numbers and volumes were scaled down two- or four-fold to accommodate to 48-well or 96-well plates. To induce activation, iDCs were stimulated with either the DC1 maturation cocktail (LPS, 1 μg/mL + IFNγ, 1,000 U/mL) for 1 (18 h), 2, 3, or 4 days or the gold standard DC (sDC) activation cocktail (TNFα, 1,000 U/mL + IL-1β, 1,000 U/mL + IL-6, 1,000 U/mL + PGE_2_ 1 μg/mL) for 18 h. Additionally, four-day activated DCs were incubated with Pan-TAM Tyrosine Kinase Inhibitor BMS-777607 (10 μM, Selleck Chemicals) and/or efferocytosis inhibitor GSK-650394 (5 μM, Sigma-Aldrich). DC culture supernatants were harvested for cytokine measurements and DCs were washed before added to a mixed lymphocyte reaction (MLR) or stained for analysis by flow cytometry.

### PBMC and T Cell Isolation

PBMCs were isolated from buffy coats obtained from healthy volunteers by density gradient centrifugation using Lymphoprep (Nycomed). Prior to being set up in a MLR, PBMCs were rested overnight in a Falcon tube positioned at a slant of 5 degrees above horizontal and the tube cap loosened in order to allow gas exchange. In selected experiments, CD4^+^ or CD8^+^ T cells were purified from PBMCs by positive selection. Briefly, according to the manufacturer's instructions, PBMCs were incubated with anti-CD4 or anti-CD8 conjugated magnetic beads in PBS with 0.5% fetal bovine serum and 2 mM EDTA. After washing the cells, they were loaded onto charged LS columns (Miltenyi Biotech) that were subsequently washed three times. The magnet was removed and labeled CD4^+^ or CD8^+^ T cells were eluted. The purity of each isolated T cell population was evaluated by flow cytometry.

### Mixed Lymphocyte Reaction

MoDCs were activated as described above. Overnight rested PBMCs were counted under a hemocytometer using the trypan blue exclusion test. DCs were harvested, washed and set up with allogeneic PBMCs or isolated T cells at a ratio of 1:10 in quadruplets in 96-well round-bottom plates to a final volume of 200 μL/well. If indicated, 10 μg/mL anti-IL-12p70 (R&D Systems) or 10 ng/mL recombinant human IL-12p70 (PeproTech) were added to the co-cultures on day zero. After 5 days, the MLR supernatants were harvested for cytokine detection.

### Cytokine Measurements

The IFNγ, IL-12p70, IL-12p40, IL-10, IL-17A, IL-23, TNFα, and IL-6 levels in the DC and MLR supernatants were measured using commercial ELISA kits (all from Invitrogen except IL-12p40 from Biolegend) according to the manufacturer's instructions. Briefly, MaxiSorp plates were coated with the respective capture antibody incubating overnight at 4°C. The remaining procedure was performed at room temperature and washing was performed between each successive step with ELISA wash buffer (PBS with 0.05% Tween 20, Sigma-Aldrich). After blocking the plates for 1 h with commercial ELISA diluent containing bovine serum, the plates were incubated with samples and standards for 2 h. Subsequently, the respective biotin-conjugated detection antibody was added and incubated for 1 h followed by addition of streptavidin-horseradish peroxidase incubating for 30 min. Color was developed using 3,3′,5,5′-tetramethylbenzidine substrate and the reaction was stopped after 10–15 min with 1M phosphoric acid. Absorption was measured at 450 nm and 570 nm on an Epoch Microplate Spectrophotometer (BioTek Instruments). The cytokine concentrations in the samples were calculated based on the resulting standard curves in Gen5 software (BioTek Instruments). The levels of IFNγ, IL-12p70, IL-12p40, IL-10, IL-17A, IL-23, TNFα, and IL-6 were detected with a lower sensitivity of 156, 7.8, 312, 94, 8, 32, 16, and 6.3 pg/mL, respectively.

### DC Staining for Flow Cytometry

Prior to staining, cells were washed in FACS buffer [PBS, 0.05% sodium azide with 0.5% bovine serum albumin (Sigma-Aldrich)]. Both during and after the staining procedure, cells were kept cold and in the dark. To be able to discriminate between live and dead cells in the subsequent analysis, cells were incubated with LIVE/DEAD Fixable Near-IR Stain (Invitrogen) according to manufacturer's instructions. DCs were additionally incubated with human IgG immunoglobulin (20 μg/mL, Kiovig Baxter) to block Fc receptors. The following fluorochrome labeled monoclonal antibodies were used for staining: anti-CD80 (L307.4, BD Pharmingen) and anti-CD86 (2331, BD Pharmingen). Cells were fixed in 1% paraformaldehyde and acquired within 5 days on a BD FACSCanto II (BD Biosciences). Unstained DCs were included in the analysis and used for calculating the mean fluorescence intensity (MFI) ratios between stained and unstained samples. Single-stained BD ComBead Plus compensation particles (BD Biosciences) were used for compensation. Data were analyzed using BD FACSDiva software version 8.0.2 (BD Biosciences).

### DC Apoptosis Detection

DCs were washed in FACS buffer and resuspended in Annexin V binding buffer (BioLegend). Subsequently, cells were stained with FITC-conjugated Annexin V and 7-AAD (both BioLegend) for 15 min at room temperature and acquired immediately after on a BD FACSCantoII.

### Protein Screening Using Proximity Extension Assay (PEA)

The Proximity Extension Assay (PEA) technology is a 92-plex immuno-PCR method enabling large-scale multiplex screening of protein biomarkers in targeted protein panels. The PEA technology uses two paired oligonucleotide-conjugated antibodies as probe for each protein. When a pair of probes recognize and bind to a common target protein (dual binding), the DNA oligonucleotides are brought in proximity and can hybridize, allowing enzymatic DNA polymerization to produce a new amplifiable DNA molecule (amplicon). The amplicon is subsequently detected and quantified using microfluidic real-time PCR. The technology reports relative protein values, allowing comparison between groups of samples. The samples were thawed at 4°C, vortexed and spun down at 400 × g at 1 min. One microliter of supernatant was added to 3 μL incubation mix in a 96-well plate, with each well containing 92 pairs of probes (A- and B-probes), incubation solution and incubation stabilizer. The plate was incubated at 4°C overnight (16–22 h). The next day 96 μL extension mix containing PEA solution, PEA enzyme and PCR polymerase was added to each well. The plate was vortexed thoroughly, spun down and transferred to the thermal cycler (Veriti 96 well Thermal Cycler, Applied Biosystems) for an initial DNA extension at 50°C for 20 min followed by 17 cycles of DNA amplification. The final step in the protocol quantifies the DNA amplicons for each protein using the Fluidigm Biomark system (Fluidigm, South San Francisco, CA, USA). The plate with extension products was vortexed and spun down. In a new plate, 2.8 μL of each extension product was mixed with 7.2 μL detection mix consisting of detection solution, detection enzyme and PCR polymerase. A 96.96 Dynamic Array Integrated Fluidic Circuit (Fluidigm) was prepared and primed according to the manufacturer's instructions. The plate with samples was vortexed thoroughly and spun down. From the sample plate 5 μL was loaded into the right side of the primed 96.96 IFC. From the primer plate the unique primer pairs for each protein were loaded into the left side of the 96.96 IFC.

The PEA readout is a relative quantification of protein abundance measured in Normalized Protein Expression (NPX), which is an arbitrary unit on log2 scale where a high NPX corresponds to a high protein abundance. NPX-values are calculated from cycle threshold (Ct) values exported from the Fluidigm Real-Time PCR Analysis software (Fluidigm, South San Francisco, CA, USA) using NPX Manager software (Olink Proteomics, Sweden). For each assay, protein concentration detection limit (pg/mL), lower limit of quantification (LLOQ) and upper limit of quantification ULOQ) are reported in the validation data for each protein biomarker panel. The detection limit is calculated as three times the standard deviation over the background signal.

### Statistical and Biocomputational Analysis

Data were analyzed with GraphPad Prism 8 (GraphPad Software). Two-way ANOVA with Tukey's *post-hoc* or Dunnett's multiple comparisons test was performed to determine the statistical significance between groups, where ****p ≤ 0.0001, ***p ≤ 0.001, **p ≤ 0.01, *p ≤ 0.05 and ns, not statistically significant. When indicated, tests were performed on log10-transformed data to homogenize the variances of the groups. D'Agostino & Pearsons normality test was applied to test if log10-transformed data within groups followed a Gaussian distribution. Pearson's correlation test was used to test for correlations between cytokine levels in the DC supernatants.

Biocomputational analysis was performed on the PEA data using the freely available web tool ClustVis (http://biit.cs.ut.ee/clustvis/). Clustvis is written using R statistics software package version 0.10.2.1 (15). The data was visualized using principal component analysis (PCA). PC1 on the x axis was displayed vs. PC2 on the y axis. Furthermore, was a heatmap created for further visualization using the settings for clustering distance set to correlation, clustering method was average and the tree ordering shows the tightest cluster first for both rows and columns.

## Results

### Loss of IL-12p70 Associates With IL-10 Release During Long-Term Exposure to Inflammation

**Figure 1:**
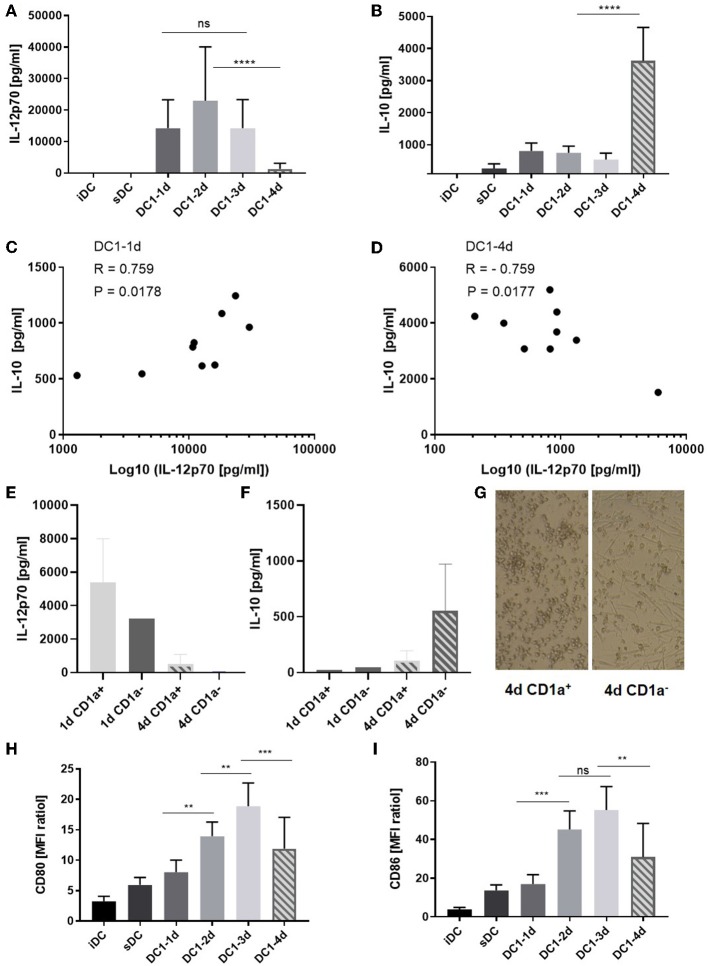
Cytokine and co-stimulatory receptor alterations during extended maturation of DCs. Immature DCs activated with the DC1 (LPS + IFNγ) cocktail were harvested after 1 (18 h), 2, 3, or 4 days (DC1-1d, DC1-2d, DC1-3d, DC1-4d). Immature DCs (iDCs) and sDCs, stimulated with the gold standard activation cocktail (TNFα + IL-1β + IL-6 + PGE_2_) for 1 day (18 h), were included for comparison. The concentrations of **(A)** IL-12p70 and **(B)** IL-10 were measured in the DC supernatants. Cumulative data are shown from five independent experiments with seven unique donors in total (*n* = 9). Bars represent mean + standard deviation. The lower limits of detection were 7.8 pg/mL for IL-12p70 and 94 pg/mL for IL-10. Two-way ANOVA with Tukey's *post-hoc* test was performed on log10-transformed data to compare DC1 groups where ****p ≤ 0.0001, ***p ≤ 0.001, **p ≤ 0.01, and ns, not statistically significant. Pearson's correlation test, where *p ≤ 0.05, was used to test for correlations between the level of IL-12p70 and IL-10 measured in the DC1 supernatants on day 1 **(C)** and day 4 **(D)** of maturation. X-axis is shown on a base 10 logarithmic scale. Levels of IL-12p70 **(E)** and IL-10 **(F)** is shown from 1- or 4-day activated CD1a^+^ and CD1a^−^ sorted fractions of iDCs (*n* = 2) and morphology of each day 4 DC1 subfraction is shown (100X magnification) **(G)**. CD80 **(H)** and CD86 **(I)** expression is shown as cumulative data for five-six donors from two-four independent experiments (*n* = 5–6). Data are presented as MFI ratios (stained sample MFI/unstained sample MFI). Two-way ANOVA with Tukey's *post-hoc* test was performed on the data to compare DC1 groups where ****p ≤ 0.0001, ***p ≤ 0.001, **p ≤ 0.01, and ns, not statistically significant. Bars represent mean + standard deviation.

Immature MoDCs were thawed on day zero and incubated in medium with GM-CSF and IL-4. When indicated, inflammatory agents were added to the DCs and on day four, all DCs and supernatants were collected and subjected to further analysis. No significant differences between the levels of IL-12p70 measured on day 1, 2, and 3 were observed (Figure 1A) and whereas the absolute levels of IL-12p70 varied from donor to donor, the kinetics were equivalent among donors (Figure S1A). A significant reduction of IL-12p70 (mean 91%, *p* < 0.0001) was observed between day 3 and 4 (Figure 1A). In contrast, IL-10 only dropped moderately between day 1 and three, followed by a six-fold increase on day 4 (*p* < 0.0001) (Figure 1B and Figure S1B). Further, while donor-specific levels of IL-12p70 and IL-10 in supernatants from 1-day activated DC1s were positively correlated (*p* < 0.05) (Figure 1C), they were inversely correlated in supernatants from 4-day activated DC1s (*p* < 0.05) (Figure 1D). This strongly suggests *de novo* production of IL-10 following long-term activation of DC1s. CD1a^+^ MoDCs have been described as main producers of IL-12p70 and allthough highly donor-dependent, typically account for about 50% of cells in fetal bovine serum-supplemented culture conditions (14). Thus, iDCs were sorted into CD1a^+^ and CD1a^−^ fractions and activated for 1 or 4 days. Interestingly, whereas CD1a^+^ 1-day activated DC1s were main producers of IL-12p70, CD1a^−^ four-day activated DC1s were main producers of IL-10 (Figures 1E,F). Strikingly, CD1a^+^ and CD1a^−^ fractions differed substantially in their morphology. Whereas, maturing CD1a^+^ DCs maintained a rounded shape, CD1a^−^ DCs seemed more adherant and had a spindle-shaped morphology (Figure 1G). Finally, phenotypic analysis of DC1s revealed that expression of costimulatory molecules CD80 and CD86 increased over the course of activation while being down regulated on 4-day activated DC1s (Figures 1H,I). This expression pattern was similar for all donors (*n* = 5) except one, where CD80 and CD86 expression further increased on four-day activated DC1s. Additional ELISA analysis of IL-12p40, IL-23, TNFα, and IL-6 from DC supernatants aligned with IL-12p70 in terms of decreasing levels observed in supernatants from four-day DC1s albeit in the case of TNFα and especially IL-6 at less striking changes (Figures S1C–F).

### Four-Day Activated DC1s Efferocytose Apoptotic DCs

**Figure 2:**
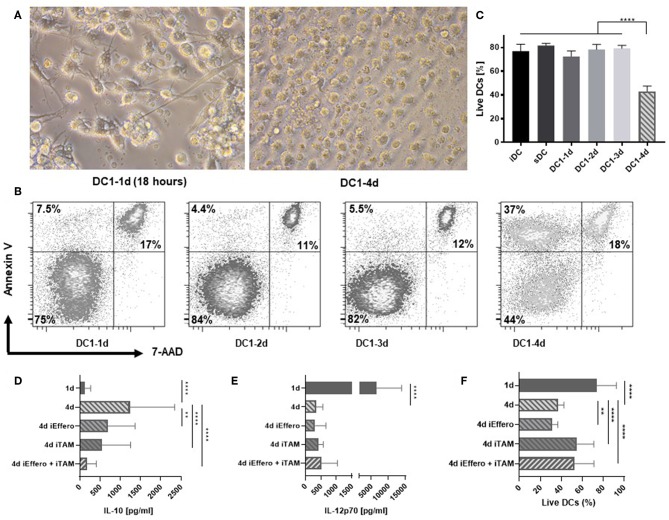
Apoptosis is induced in DCs after 4 days of activation with LPS and IFNγ. **(A)** Morphology of 1- and 4-day activated DC1s (400X magnification). Four-day activated DC1s exhibit clear signs of undergoing apoptosis such as cell shrinkage and increased amounts of cell debris. Photographs are representative of six donors (*n* = 6). **(B)** Apoptotic DCs were detected by staining with Annexin V and 7-AAD and percentages of live, pre-apoptotic, and dead cells is shown. The data is representative of three independent experiments with four donors (*n* = 4). **(C)** Viability of DCs as a LIVE/DEAD Fixable Near-IR Stain negative percentage of FSC and SSC gated cells. Four-day activated DCs were cultured in the precense of specific inhibitors targeting TAM receptor signaling (iTAM) and/or efferocytosis (iEffero). **(D)** IL-10 and **(E)** IL-12p70 was measured in the supernatants and **(F)** the viability of DCs was measured with LIVE/DEAD Fixable dye. Cumulative data are shown from two independent experiments with four donors (*n* = 4). Bars represent mean + standard deviation. Two-way ANOVA with Tukey's *post-hoc* or Dunnett's multiple comparisons test was performed on non-transformed data to compare groups (****p ≤ 0.0001, **p ≤ 0.01, and ns, not statistically significant).

We further characterized the consequences of prolonged exposure of DCs to inflammation. Interestingly, the morphological appearance of 4-day activated DC1s exhibited clear signs of apoptosis (Figure 2A). These included classical hallmarks of apoptosis, such as smaller cell size due to cellular shrinkage and increased amounts of cell debris (16). In contrast to 1-day activated DC1s, 4-day activated DC1s no longer formed extensive dendritic projections. To confirm these observations, DCs were stained with Annexin V and 7-amino-actinomycin D (7-AAD), allowing for detection of early and late apoptotic events, respectively. In contrast to all other samples, a large fraction of 4-day DC1s appeared as pre-apoptotic Annexin V positive 7-AAD negative cells, while no major differences were seen for late apoptotic (7-AAD positive) and live DCs (Figure 2B). A systematic evaluation of DCs from six donors stained with a fixable viability dye revealed a significantly (*p* < 0.001) reduced amount of live 4-day activated DC1s out of total cellular events (Figure 2C). Thus, apoptosis associates with major changes in the IL-12p70 and IL-10 concentrations in the DC culture supernatant following long-term exposure to LPS and IFNγ. To further understand why cytokine levels changed dramatically on day 4 of activation, the influence of efferocytosis and TAM receptors was evaluated. Interestingly, dual blockade potently inhibited the release of IL-10 on 4-day activated DC1s (Figure 2D) but did not impact levels of IL-12p70 (Figure 2E). Furthermore, while TAM receptor blocade reduced the induction of apoptosis in 4-day activated DC1s, single blockade of efferocytosis seemed to enhance the induction of apoptosis (Figure 2F). Indeed, previous studies have linked efferocytosis to production of immunosupressive molecules such as IL-10 and PGE_2_ (17), but this has not been documented over a time course of DC activation.

### Long-Term Activated DCs Induce Progressively Lower IFNγ Secretion From Alloreactive T Cells

**Figure 3:**
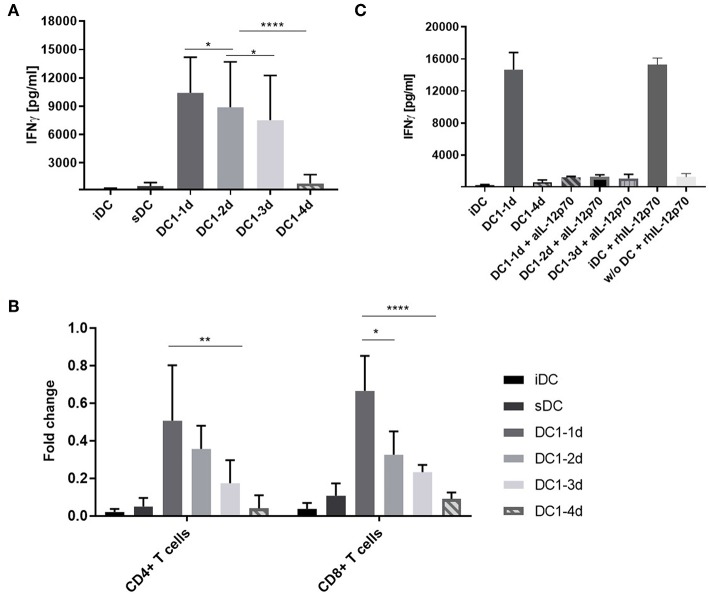
IFNγ release from co-cultures with long-term activated DCs is reduced. **(A)** DCs were activated with the DC1 maturation cocktail (LPS and IFNγ) for 1, 2, 3, or 4 days (DC1-1d, DC1-2d, DC1-3d, DC1-4d) prior to being set up in co-culture with allogeneic PBMCs at a 1:10 ratio. After 5 days, IFNγ release was measured in the MLR supernatants with a lower limit of sensitivity of 156 pg/mL. Co-cultures with iDCs and sDC were included for comparison. Cumulative data are shown from five independent experiments with nine unique donor pairs in total (*n* = 9), consisting of seven DC donors and five PBMC donors. All experiments were performed in quadruplicates. Bars represent mean + standard deviation. Two-way ANOVA with Tukey's *post-hoc* test was performed on log10-transformed data to compare DC1 groups (****p ≤ 0.0001, **p ≤ 0.01, *p ≤ 0.05, and ns, not statistically significant). **(B)** Same as **(A)**, except isolated CD4^+^ and CD8^+^ T cells were used as responder cells instead of PBMCs. Data from three donors (*n* = 3) are normalized to highest value of IFNγ release within each donor. Bars represent mean + standard deviation. Two-way ANOVA with Tukey's *post-hoc* test was performed on normalized data to compare DC1 groups (****p ≤ 0.0001, **p ≤ 0.01, *p ≤ 0.05). The lower limit of IFNγ detection was 39 pg/mL. **(C)** Co-cultures were set up as in **(A)** and supplemented with anti-IL-12p70 or recombinant human IL-12p70. Recombinant human IL-12p70 was also added to cultures of PBMCs without DCs (w/o DC). After 5 days, IFNγ release was measured. Cumulative data are shown from one experiment with three unique donors (*n* = 3). Bars represent mean + standard deviation.

To examine if pro-longed exposure of DCs to inflammation would affect the functionality and ability to induce cell activation, DC1s were stimulated with allogeneic peripheral blood mononuclear cells (PBMCs) in a MLR setup. After 5 days, IFNγ was quantifyed as a measure of alloantigen-specific cell activation. Extending DC1 activation for 2 or 3 days prior to co-culture resulted in a progressive reduction in IFNγ (*p* < 0.05). Notably, 4-day activated DC1s induced a nine-fold lower IFNγ response (*p* < 0.001) compared to three-day activated DC1s (Figure 3A). ELISA measurements of IL-12p70, IL-10 and IL-17A revealed similar response-patterns between sample groups albeit at much lower absolute levels (Figures S2A–C). A comparable and dose-dependent pattern was observed, when the number of DCs in co-culture was titrated down from 10.000 to 5.000 and 2.500 DCs (Figure S2D).

Bulk PBMCs are easily obtained and contain several lymphocyte fractions as well as myeloid cells. Thus, isolated fractions of allogeneic CD4^+^ or CD8^+^ T cells were used in an equivalent five-day co-culture setup. Indeed, similar IFNγ response patterns as with PBMCs were observed for both cell types (Figure 3B). This suggested that both T cell populations were contributing to the IFNγ release observed in Figure 3A.

In a previous study it was shown that DCs activated with LPS and IFNγ for 21–27 h could regain IL-12p70 secretion when activated by T cells in co-culture (18). To examine the functional importance of IL-12p70 in our setup, a neutralizing antibody against IL-12p70 or recombinant human IL-12p70 was added to the co-cultures. Addition of anti-IL-12p70 to co-cultures with 1-, 2-, or 3-day activated DC1s resulted in a 12-fold decrease in the IFNγ measured in the MLR supernatants after 5 days (Figure 3C). This confirmed the role of IL-12p70 as an IFNγ response inducer (19). In line, addition of recombinant IL-12p70 to immature DCs but not cultures without DCs, increased the release of IFNγ to the level observed with 1-day activated DC1s. Conclusively, long-term activation (4 days) of DC1s impair their functionality and lack of IL-12p70 secretion results in non-functional T cells devoid of IFNγ.

### Prolonged Stimulation With LPS and IFNγ Induces Release of Multipe Inflammatory Proteins

**Figure 4:**
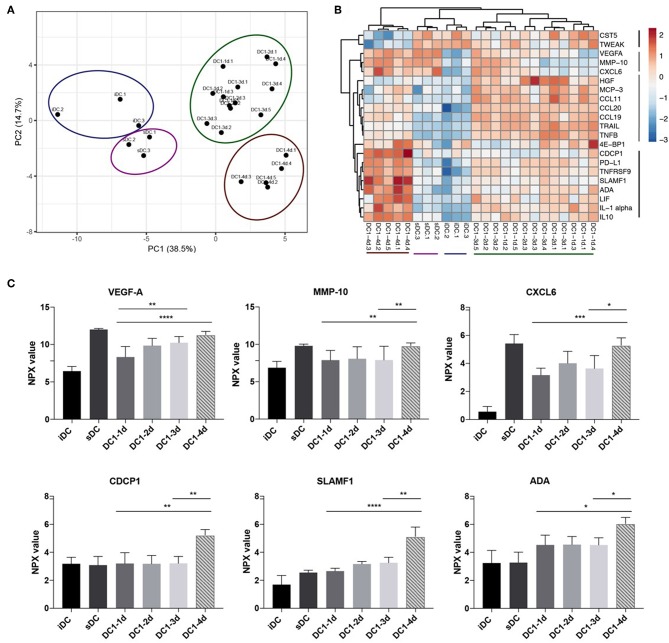
Long-term exposure to inflammation induces differential cytokine patterns in dendritic cells. **(A)** Principle component analysis of 50 detectable proteins measured in culture supernatants from DCs activated with the DC1 activation cocktail (LPS and IFNγ) for 1, 2, 3, or 4 days (DC1-1d, DC1-2d, DC1-3d, DC1-4d). Immature DCs (iDCs) and sDCs, stimulated with the gold standard activation cocktail (TNFα + IL-1β + IL-6 + PGE_2_) for 1 day (18 h), were included for comparison. Supernatant from three-five donors was included in the analysis (*n* = 5 for 1-, 3-, and 4-day activated DCs). **(B)** Dendrogram depicting hierarchal clustering and heat map analysis of normalized levels of 21 proteins found by two-way ANOVA to significantly differ between five matched samples from 1-, 3-, and 4-day activated DC1s. **(C)** Six proteins that besides IL-10 was also strongly upregulated in 4-day activated DC1s. Two-way ANOVA with Tukey's *post-hoc* test was performed to compare DC1 groups where ****p ≤0.0001, ***p ≤0.001, **p ≤0.01, *p ≤0.05.

Finally, it was investigated if global changes in protein release from the stimulated DCs accompanied the decrease in IL-12p70 and increase in IL-10 observed on day 4. Undiluted DC supernatants were analyzed in a multiplex immunoassay (Proximity Extension Assay, PEA) with a panel of 92 inflammation-related proteins (Proseek Multiplex Inflammation I). Five paired supernatants from each of the DC1 groups, one-day, three-day and four-day (DC1-1d, DC1-3d, and DC1-4d), as well as three supernatants from two-day DC1s (DC1-2d), were analyzed. Three matched samples from immature DCs (iDCs) and sDCs and a control medium sample were also analyzed. Matched ELISA and PEA data from four proteins (IL-10, TNFα, IL-6, and IL12p40) was used to compare the two methods. Overall, data within the lower and upper limit of quantitation (LLOQ and ULOQ) was comparable and highly correlated as seen for IL-10 and TNFα (Figures S3A,B). However, due to saturation, PEA measurements of IL-6 and IL-12p40 above ULOQ did not correlate with ELISA results (Figures S3C,D). For this reason, and because PEA was optimized to measure proteins in plasma and serum, a total of 17 proteins were excluded from analysis due to saturation (values above ULOQ) on a subset of samples. Furthermore, in the case of 20 proteins, more than 80% of samples were below limit of detection and was therefore excluded. To avoid over-interpretation, five additional proteins with values around limit of detection were excluded leaving 50 proteins for interpretation. Principle component analysis (PCA) was employed to assess relatedness between samples on normalized sample data. All six control samples of iDCs and sDCs clustered separately from all DC1 samples. Additionally, another cluster of all 4-day DC1 samples was separate from the remaining DC1 samples (Figure 4A). To assess significant changes in protein levels during prolonged stimulation with LPS and IFNγ, the matched samples from five donors were analyzed by two-way ANOVA comparing one, 3 and 4 days of stimulation. Significant variations (*p* < 0.05) were found for 21 out of 50 proteins measured. To further understand how these 21 proteins differed between samples, a heatmap was generated displaying hierarchical clustering of normalized values from all samples. Four groups of proteins were found of which two groups included proteins upregulated in four-day DC1s (Figure 4B). Interestingly, one of these groups was also induced by sDC activation. Aside from IL-10 (Figure S3E), six other proteins were strongly upregulated (*p* < 0.01) in four-day DC1s including three proteins co-induced by sDCs (VEGF-A, MMP-10 and CXCL6) as well as CDCP1, SLAMF1, and ADA exclusively induced in 4-day DC1s (Figure 4C). The difference in cytokine expression profiles of the different activation stages shows a striking change from 2- to 4-day DC1s (Figure 4B). In conclusion, prolonged exposure to inflammation correlated with a higher release of several inflammation-related proteins from DCs, which was significantly different from short-term activated DCs.

## Discussion

Taken together, we show that long-term exposure to LPS and IFNγ affects the phenotype, cytokine production, cell activating capacity and viability of human MoDCs. Most strikingly, 4-day activated DC1s undergo apoptosis concomitant with a profound induction of efferocytosis and TAM receptor-dependent IL-10 and an inversely associated drop in IL-12p70 as well as other pro-inflammatory cytokines. Most notably, we show that upon long-term inflammation, IL-10 seems to be primarly produced by the CD1a^−^ subfraction of DC1s whereas levels of IL-12p70, initially produced upon short-term activation by primarily CD1a^+^ DC1s, is now inversible correlated. Further work is needed to elucidate if these subfractions are actively interacting e.g., during the process of efferocytosis or if transcriptional programs governing early IL-12p70 and late IL-10 release are independent processes. The striking difference in morphology is also noteworthy as spindle-shaped MoDCs is a well-known *in vitro* phenomena dependent on activation with IFNγ (20). In our experience, fraction of spindle-shaped DCs seems donor-dependent and to our knowledge have not been dictomized between CD1a^−^ and CD1a^+^ subfraction in prior studies. Secretion of anti-inflammatory cytokines, such as IL-10, is one of several ways, by which the immune system can limit an inflammatory response, induce tolerance and thereby inhibit reactivity toward self (21). IL-10 is produced by many immune cells of both the innate and adaptive immune system. In DCs, activation with LPS and other TLR ligands induces concurrent production of IL-12 and IL-10, the latter serving as a myeloid checkpoint to avoid immunopathology (22). IL-10 has an inhibitory function on IL-12, as it down-regulates the transcription of both *p40* and *p35* (23) and autocrine production of IL-10 down-regulates the expression of surface co-stimulatory molecules and limits production of IL-12 and TNFα (22). However, our study appears to show that such autocrine signaling does not occur in this prolonged DC activation setting possibly due to lack of re-activating signals e.g., CD40L stimulation (18) allowing *de novo* synthesis of IL-12p70. Thus, additional yet unkown mechanism of actions may account for the subtle drop in proinflammatory cytokine levels that accompany the release of IL-10. While, TAM-receptor blockade increased viability and reduced levels of IL-10, blocking efferocytosis did not improve viability of long-term activated DCs. Interestingly, however our data support the combination of both drugs for efficient blockade of IL-10 and thus may add to new data suggesting benefit from combining TAM-receptor blockade and PD-1 blockade in syngenic mouse tumor models (24).

Programmed cell death of DCs is a regulatory mechanism, which can be promoted by several factors, such as IL-10 and LPS (25). Along with being associated with impaired DC activation and function, the tumor microenvironment has also been shown to induce apoptosis in DCs (26). Interactions with apoptotic cells can induce differentiation of immature DCs into tolerogenic DCs that secrete IL-10 and induce regulatory T cell (Treg) differentiation. An early study showed that incubation of monocytes with apoptotic cells during activation with LPS led to increased secretion of IL-10 and decreased secretion of TNFα and IL-1β (27). Later, it was described that phagocytosis of apoptotic DCs by immature DCs primes them to become tolerogenic and promote development of induced Tregs from naïve T cells (28).

Here, we found that IFNγ was progressively lost as exposure of the DC1s to inflammatory stimuli was extended from 1 up to 4 days prior to MLR co-culture. Particularly, the IFNγ response was reduced nine-fold in co-cultures of allogeneic PBMCs with DCs activated for four compared with 3 days. Antibody-mediated blockade of IL-12p70 resulted in a similar reduction in IFNγ, verifying the functional relevance of DC-secreted IL-12p70 in induction of IFNγ.

Finally, we employed multiplex analysis of DC supernatants to further understand the changes induced in DCs following long-term inflammation. We took advantage of ClustVis, a freely available web tool for visualizing clustering of multivariate data using PCA and heatmaps (15). We found that PCA analysis separated 4-day activated DC1s from the remaining 1, 2, or 3-day activated DC1s. Furthermore, all five samples from 4-day activated DC1s formed a separate cluster from remaining DC1s in the heatmap, and expressed increased levels of eight proteins (CDCP1, PD-L1, TNFRSF9, SLAMF1, ADA, LIF, IL-1α, IL-10). These proteins are involved in a broad range of functionalities related to apoptosis, cancer and conditions of chronic inflammation. Thus, CDCP1 is a novel marker of the most aggressive human triple-negative breast cancers (29). PD-L1 is a well-known cancer immune checkpoint that when silenced in DCs augments expansion and function of antigen-specific T cells (30). SLAMF1 is overexpressed in myeloid cells from patients with Crohn's disease and in T lymphocytes from patients with rheumatoid arthritis (31). In regards to apoptosis, TNFRSF9 or 4-1BB acts as a survival factor in DCs (32) and the enzyme ADA abrogates the anti-inflammatory effects of adenosine present in apoptotic supernatants (33). IL-1α is a proinflammatory cytokine induced during pyroptosis, involving plasma-membrane permeabilization of macrophages and DCs upon LPS stimulation and downstream caspase-1 and inflammasome activation (34). In conclusion, proteins upregulated in 4-day activated DC1s identify features that associates with induction of apoptosis and associates with conditions of cancer and chronic inflammation.

Furthermore, another cluster of proteins comprising VEGF-A, MMP-10 and CXCL6 was upregulated in 4-day activated DC1s as well as non-functional sDCs activated by PGE_2_, TNFα, IL-1β, and IL-6. VEGF-A confined to lymph nodes is primarily produced by DCs (35) and activation by anti-inflammatory molecules such as calcitriol, PGE_2_, or IL-10 has been shown to induce production of VEGF-A (36). Interestingly, PEA analysis of plasma from metastatic melanoma patients treated with immune checkpoint inhibitors showed association between both VEGF-A and IL-10 and shorter progression free survival (37). In a vaccination setting, MMP-10 was critical for *in vivo* tolerance and release of IL-10 and TGFβ1 upon repeated cutaneous exposure to the TLR7 agonist, Imiquimod (38). CXCL6 is a neutrophil chemoattractant associated with chronic inflammation and predominantly produced in mesenchymal cells stimulated with IL-1β and counteracted by IFNγ (39). Conditions of IL-1β and hypoxia also promotes CXCL6 in cell lines from small cell lung cancer (40). In conclusion, VEGF-A, MMP-10, and CXCL6 share features of non-functional, anti-inflammatory tumor-promoting markers that may be induced upon selected stimuli. Prior data had already documented a skewed chemokine pattern, in which DC1 primarily produce abundant levels of pro-inflammatory CXCL10 and CCL5 as opposed to sDCs that mainly secreted Treg attracting CCL22 (41). These new findings thus further underscore the disadvantages of using sDCs for cancer vaccination approaches. Instead we suggest that short-term activated DC1s represent a desired state of acute inflammation, whereas sDCs and long-term activated DC1s represent a state of chronic inflammation often found during chronic viral infection and cancer.

To summarize, long-term activated DC1s dramatically changed their cytokine secretion profile toward a tumor-promoting and anti-inflammatory phenotype. After 4 days of activation, DC1s became apoptotic and non-functional, highlighting the difference between acute and chronic states of inflammation.

## Data Availability Statement

The datasets generated for this study are available on request to the corresponding author.

## Author Contributions

MH conceived the idea behind the study. LC and MH designed the experimental setup. LC and OL-A performed the experiments with the help from AO. LC, OL-A, and MH analyzed and interpreted the data. LC, OL-A, and MH prepared figures. MH and LC wrote the manuscript. IS provided the funding and together with MC critically reviewed the manuscript.

### Conflict of Interest

OL-A is employed by company Immunitrack ApS. The remaining authors declare that the research was conducted in the absence of any commercial or financial relationships that could be construed as a potential conflict of interest.
