# Supplementary material for: Long-Term Exposure to Inflammation Induces Differential Cytokine Patterns and Apoptosis in Dendritic Cells
Source: Front Immunol. 2019 Nov 21;10:2702. doi: 10.3389/fimmu.2019.02702 (PMC6882286; doi:10.3389/fimmu.2019.02702)
Supplement: Supplementary file 1 [file Data_Sheet_1.pdf]

Supplementary Figures

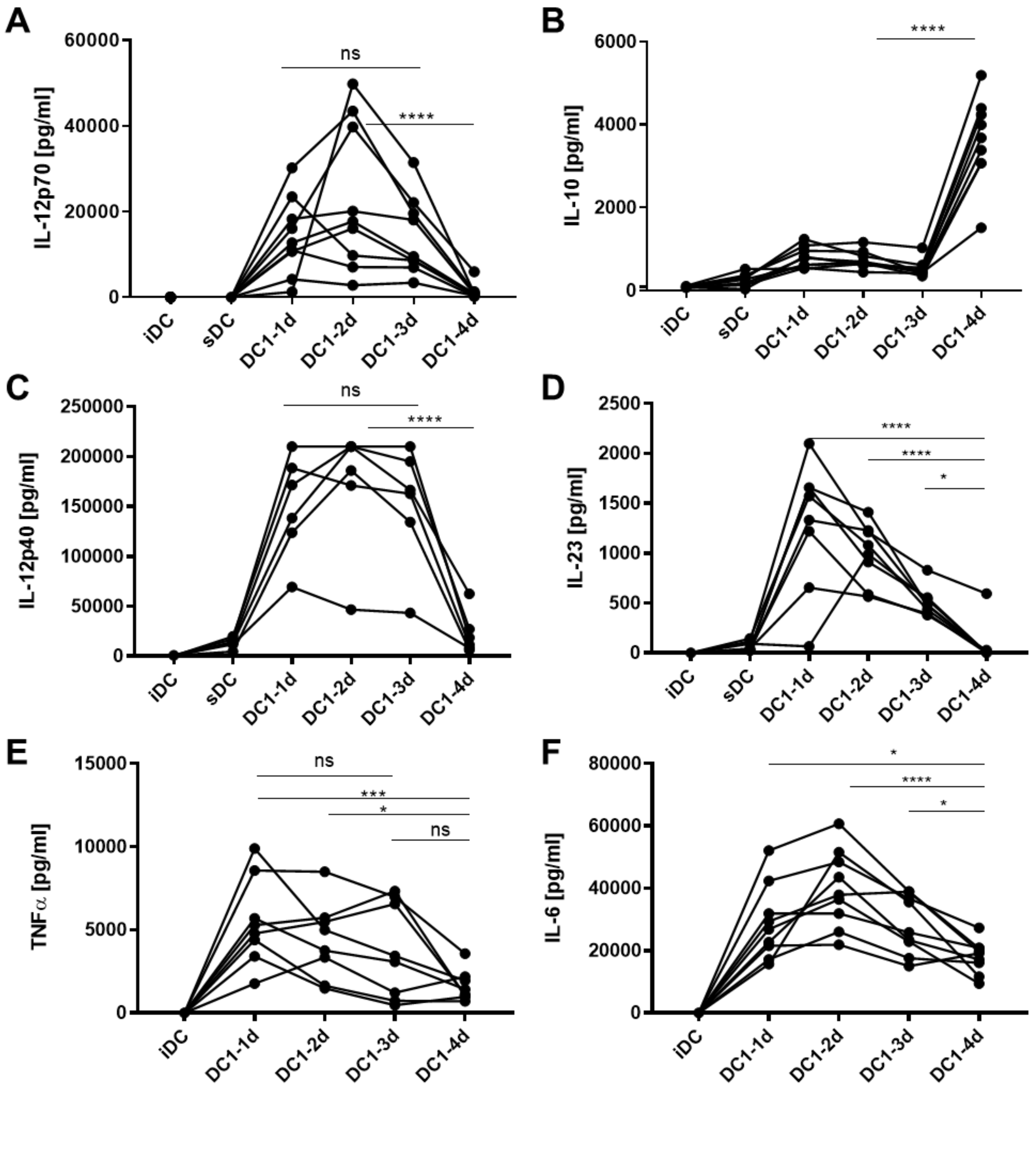

Supplementary Figure 1. ELISA analysis of IL-12p70, IL-10, IL-12p40, IL-23, TNFα and IL-6 in DC supernatants. DCs activated with the DC1 (LPS + IFNγ) cocktail were harvested after one (18 hours), two, three or four days (DC1-1d, DC1-2d, DC1-3d, DC1-4d). Immature DCs (iDCs) and

sDCs, stimulated with the gold standard activation cocktail (TNFα + IL-1β + IL-6 + PGE_2) for one day (18 hours), were included for comparison. The concentrations of (A) IL-12p70, (B) IL-10, (C) IL-12p40, (D) IL-23, (E) TNFα and (F) IL-6 were measured in the DC supernatants. Cumulative data are shown from several independent experiments with at least six unique donors (n=8). Bars represent mean + standard deviation. Two-way ANOVA with Tukey's post hoc test was performed to compare DC1 groups where ****p ≤0.0001, ***p ≤0.001, **p ≤0.01, *p ≤0.05 and ns = not statistically significant.

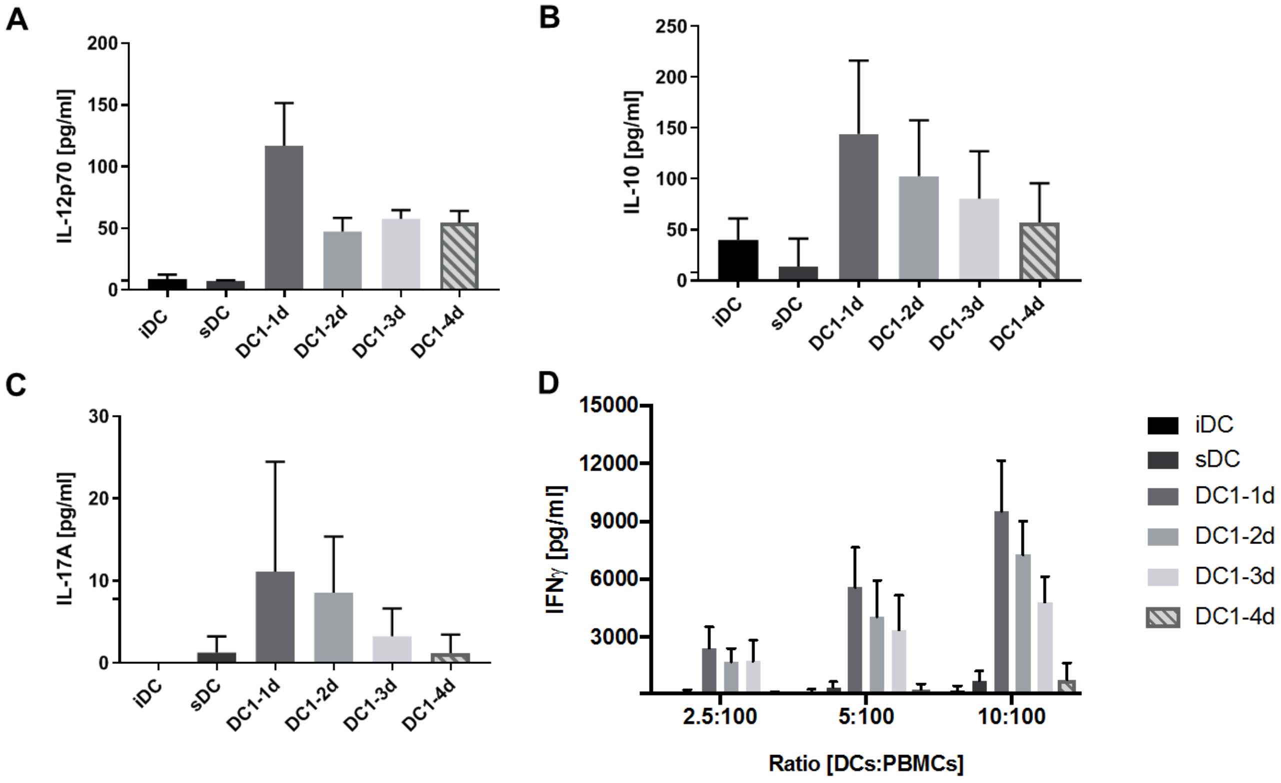

Supplementary Figure 2. ELISA analysis of IL-12p70, IL-10 and IL-17A and IFNγ release in MLR supernatants. DCs were activated with the DC1 maturation cocktail (LPS and IFNγ) for one, two, three or four days (DC1-1d, DC1-2d, DC1-3d, DC1-4d) prior to being set up in co-culture with allogeneic PBMCs (A-C) at 10:100 ratios and D) at 2.5:100, 5:100 and 10:100 ratios, showing that IFNγ release is dependent on the ratio of DCs to PBMCs. After five days, IL-12p70 (n=3), IL-10 (n=8), IL-17A (n=8) and IFNγ (n=3) release was measured in the MLR supernatants.. Co-cultures with iDCs and sDC were included for comparison. Cumulative data are shown from independent experiments with unique donor pairs. All experiments were performed in quadruplicates. Bars represent mean + standard deviation.

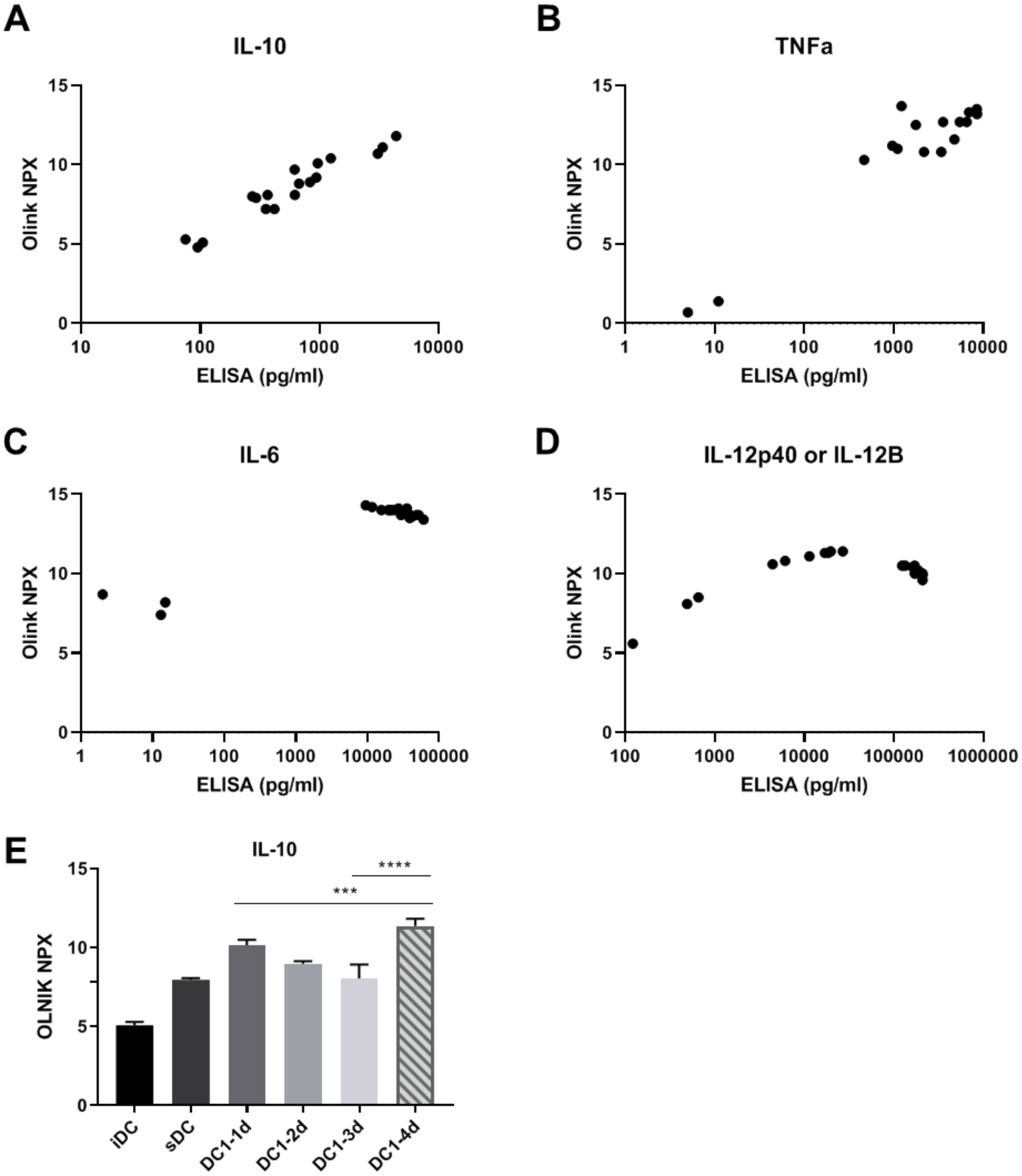

Supplementary Figure 3. Comparison of ELISA and PEA for measurement of cytokines in DC supernatants. Matched ELISA (X-axis) and PEA (Y-axis) sample data of all six sub-types of DC supernatants were compared to evaluate consistency between the two methods. The sample material included measurements of four proteins (A) IL-10, (B) TNFα, (C) IL-6 and (D) IL-12p40/IL-12B, where E) shows OLINK NPX data for IL-10 for iDC, sDC and DC1-1d, 2d, 3d and 4d. Whereas measurements of IL-10 and TNFα where highly correlated, data from IL-6 and IL-12p40 indicated saturation accounting for the inaccurate measure obtained by PEA measurements.
